# Sterilization of free-ranging female capybaras (*Hydrochoerus hydrochaeris*): a comparison between two surgical techniques

**DOI:** 10.1590/1984-3143-AR2024-0053

**Published:** 2024-09-23

**Authors:** Fabiana Morse Gosson Jorge, Fernanda Battistella Passos-Nunes, Pedro Nacib Jorge-Neto, Flavia Maria Pia Montenegro Donoso, Mariana Passos Nunes, Alexia Gazzola Steiner, Marcelo Bahia Labruna, Ana Clara Kohara Roman, Marilú Cristofoli, Mayla Magalhães de Oliveira Alcobaça, Cristiane Schilbach Pizzutto, Antonio Chaves de Assis

**Affiliations:** 1 AZ Nunes & Cia, Itu, SP, Brasil; 2 Departamento de Cirurgia, Faculdade de Medicina Veterinária e Zootecnia, Universidade de São Paulo, São Paulo, SP, Brasil; 3 Instituto Reprocon, Campo Grande, MS, Brasil; 4 Departamento de Reprodução Animal, Faculdade de Medicina Veterinária e Zootecnia, Universidade de São Paulo, São Paulo, SP, Brasil; 5 Departamento de Medicina Veterinária Preventiva e Saúde Animal, Faculdade de Medicina Veterinária e Zootecnia, Universidade de São Paulo, São Paulo, SP, Brasil

**Keywords:** contraception, mini-laparotomy, population control, wildlife, Brazilian spotted fever

## Abstract

This study evaluated two surgical sterilization techniques in free-ranging female capybaras (*n* = 21). The first group underwent uterine horn ligature (HL; *n* = 11), while the second was subjected to partial salpingectomy (S; *n* = 10). We assessed total operative time, incision length, the ease of identifying reproductive structures, the adequacy of exposure for surgical performance through flank or midline approaches, and the extent of abdominal viscera manipulation for each method. The HL method emerged as faster, with an average operative time difference of 16 minutes. In the S group, a flank mini-laparotomy over the ovarian topography facilitated easy exposure of the ipsilateral ovary and uterine tube, enabling ligature and partial resection of the uterine tube but not the uterine horn exposure. However, accessing the contralateral uterine tube without a bilateral incision was impractical, thus prolonging the total operative time due to the need for patient repositioning and new antisepsis procedures. Conversely, a post-umbilical approach for the HL method necessitated only one mini-laparotomy incision, offering ample uterine exposure for hysterotomy in pregnant females. Both methods involved minimal abdominal viscera manipulation and resulted in no fatalities or postoperative complications. Although direct comparison is limited by the distinct sterilization techniques and surgical approaches, this study underscores the challenges and surgical access of each method. Our findings endorse the HL technique as an effective contraception method for female capybaras to prevent the birth of seronegative offspring that could amplify *Rickettsia* sp., the causative agent of Brazilian spotted fever.

## Introduction

Capybaras, rodents that are widely distributed in South America (Abreu Bovo et al., 2016), are known as significant amplifying hosts for Brazilian Spotted Fever (BSF) (Miranda et al., 2011; Souza et al., 2015; Ramírez-Hernández et al., 2020), a tick-borne disease transmitted by the *Amblyomma sculptum* vector (Szabó et al., 2013; Ramírez-Hernández et al., 2020) and caused by the intracellular bacterium *Rickettsia rickettsii* (Sangioni et al., 2005; Souza et al., 2015). Without timely treatment, it can lead to a mortality rate as high as 85% (Araújo et al., 2015); therefore, immediate notification to health authorities is mandated in Brazil upon disease detection (De Oliveira et al., 2016).

In BSF-endemic areas, Passos Nunes (2019) has demonstrated a correlation between the reduction of the capybara population and a subsequent decrease of *Amblyomma sculptum* tick populations. As a preventive strategy against BSF, environmental authorities in São Paulo state (Brazil) (São Paulo, 2016) recommend the implementation of reproductive management practices for capybaras. This approach aims to prevent the birth of seronegative capybaras, which could potentially amplify the transmission of the bacteria to approximately 25% of the ticks that parasitize them during a bacteremia period of 10 to 14 days. After this period, they become immune and no longer participate in the BSF transmission cycle, underscoring the importance of maintaining sterilized capybaras in BSF-endemic areas (Souza et al., 2009). Contraceptive methods previously explored for capybaras include surgical sterilization techniques and reversible immunocontraceptive vaccines, validated in male capybaras (Rosenfield et al., 2019b) but not commercially available in Brazil.

Despite this, literature on the surgical sterilization of female capybaras remains limited. Uterine tube ligature in female capybaras through a left flank approach using a surgical seal has been described by Ferraz (2013) and Rodrigues et al. (2017). Passos Nunes et al. (2020) reported a ventral abdominal approach utilizing nylon suture thread. In contrast, Yanai et al. (2022) performed a partial uterine tube occlusion utilizing titanium clamps and a minimally invasive technique involving a bilateral mini-laparotomy. Alternatively, Passos-Nunes et al. (2022) introduced a uterine horn ligature via mini-laparotomy with a single surgical incision, employing a post-umbilical approach that minimizes postoperative complications and ensures adequate exposure of both uterine horns. This innovative method also facilitates hysterotomy in pregnant females when necessary (Passos-Nunes et al., 2024), which is crucial in areas where the absence of newborns is essential to mitigate BSF transmission (Passos-Nunes et al., 2022).

Mini-laparotomy, a technique characterized by an incision smaller than 5cm (Penido et al., 2024), offers an alternative to eliminate the need for a video laparoscopy, which requires specialized training and equipment. This technique, borrowed from human medicine, has found its application in veterinary practices, including canine ovariohysterectomy (Reece et al., 2012) and South American coatis uterine tubal ligation (Penido et al., 2024). However, specific details regarding the incision size in these contexts still need to be available.

In dealing with wildlife, especially *in situ* conditions, it is paramount to prioritize the animal's physiological and behavioral well-being. Good management practices are essential to minimize stress (Pizzutto and Jorge-Neto, 2023) and safeguard animal welfare. It includes ensuring that both the capture and surgical procedures comply with the five domains for qualitative assessment and animal welfare monitoring (Mellor et al., 2020), thereby preventing the induction of adverse experiences. An effective anesthetic protocol is critical in this regard, with agents like ketamine (Gruber and Reed, 1968; Nedaei et al., 2009; Jorge-Neto et al., 2023) being favored for their ability to induce retrograde amnesia (Araujo et al., 2021), thereby mitigating the stress of the surgical experience. The potential for anesthetic reversal further enhances this approach by reducing the duration of post-anesthetic handling, contributing to the overall welfare of the animal (Araujo et al., 2018).

The goal of minimizing the duration of both the surgical procedure and anesthesia (Passos-Nunes et al., 2022) is driven by the need to reduce the risk of intraoperative and postoperative complications (Ando et al., 2005; Hardy et al., 2014; Cheng et al., 2017; Duchman et al., 2017; Bohl et al., 2018). Minimally invasive techniques that utilize smaller incisions facilitate quicker recovery (Passos Nunes, 2023) and prompt release of the animals back into their natural habitat post-procedure.

Techniques such as uterine occlusion/resection or ligature of the uterine horn present viable options for sterilizing female capybaras. While uterine tube occlusion/resection is a well-documented method for sterilization in both humans and animals, the ligature of the uterine horn is a more recent alternative. This technique offers significant advantages, including broad uterine exposure and requiring only a single incision. It is particularly suitable for areas where controlling vector hosts for diseases like Brazilian spotted fever (BSF) is critical.

The objective of the present study, therefore, was to conduct a comparative evaluation of two surgical methods for sterilization of female capybaras: the “Passos Nunes” uterine horn ligature (Passos-Nunes et al., 2022) and the partial salpingectomy. Through this comparative analysis, the study seeks to provide valuable insights into these techniques' efficacy, safety, and practicality, contributing to the broader goals of wildlife management and disease control in BSF-endemic areas.

## Methods

This study was conducted with *in situ* capybaras inhabiting private areas in three different municipalities of São Paulo State (Brazil). The present study had authorization for *in situ* population management issued by SISBIO / ICMBio / MMA under no. 79881-2 (Louveira – SP), and by DeFau / SEMIL / SP under no. 0000055329 (Salto – SP) and no. 0000069455 (Atibaia – SP). It was approved by the Ethics Committee on Animal Use of the School of Veterinary Medicine and Animal Science of the University of São Paulo (CEUA/FMVZ) for the protocol nº. 3990010822. Under protocol A4C6059, the genetic heritage accessions of the animals were recorded in Brazilian National System for the Management of Genetic Heritage and Associated Traditional Knowledge (SISGEN).

### Animals and experimental design

Free-living capybaras were captured, and exclusively females were chosen for the study. Each captured animal underwent a visual assessment to determine its sex. Male capybaras were allocated to a separate research project and underwent deferentectomy procedures. An abdominal ultrasound (Logiq E Vet, GE Health Care, with micro-convex transducer, 7.5 MHz) was utilized to assess the captured females, and those that were pregnant were excluded from this study. A total of 21 females were selected, which underwent surgical sterilization. The animals were randomly allocated into two distinct groups: the “Passos-Nunes” uterine horn ligature group (HL; *n* = 11) and the partial salpingectomy group (S; *n* = 10).

### Capture, anesthesia, and medication

**Figure 1:**
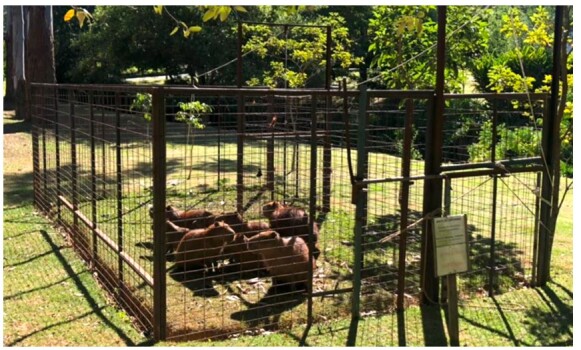
Cage trap used for passive capture following AZ Nunes Enterprises model. Photo: AZNunes LTDA.

**Figure 2:**
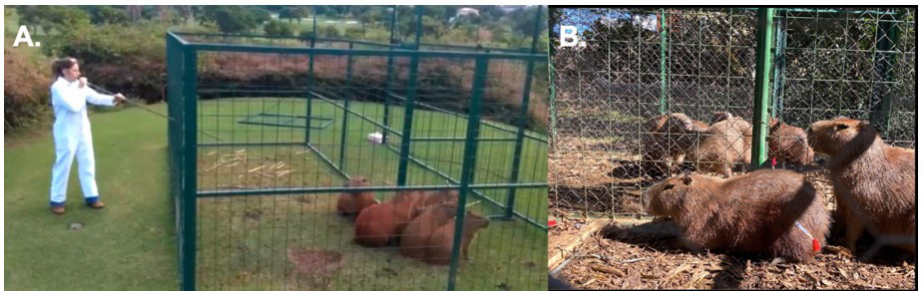
Capybaras were anesthetized with blowpipe darts containing anesthetics (A) and monitored during the anesthetic latency period (B). Photo: AZNunes LTDA.

The capture of the free-living animals was performed according to previously described by Passos-Nunes et al. (2020), where animals were baited with sugar cane or banana tree leaf in cage trap (Figure 1). After 12-hour fasting, the trapped animals were restrained using anesthetic darts fired with a blowpipe (Figure 2) containing ketamine (5 to 10 mg/kg, im; Ketalex, Venco, PR, Brazil) and xylazine (0.5 to 1.0 mg/kg, im; Sedalex, Venco, PR, Brazil) (Passos Nunes et al., 2020; Passos-Nunes et al., 2022). The anesthetic plan was assessed through the absence of oculo-palpebral and interdigital reflexes and response to surgical stimuli. Additionally, 2% lidocaine hydrochloride (0.1 mg/kg, subcutaneous infiltration; Xylestesin, Cristália, SP, Brazil) was administered locally at the incision site and topically over the uterine horns or tubes. After all the procedures, anesthesia was reversed using yohimbine hydrochloride (0.125 to 0.2 mg/kg, sc; Reset, Botupharma, SP, Brazil).Prior to surgery, meloxicam (0.6 mg/kg, sc; Maxican 2%, Ourofino, SP, Brazil) and a single dose of morphine sulfate (0.3 mg/kg, im; Hipolabor, MG, Brazil) were used for additional peri-operative analgesia. All animals also were treated with antibiotics (0.1 mL/kg; im; Agrovet 5,000,000 with procaine benzylpenicillin 3,750,000 I.U.; potassium benzylpenicillin 1,250,000 I.U.; streptomycin sulfate 2.0 g per 15 mL; Elanco, SP, Brazil) and antiparasitic (Doramectin, 0.2 mg/kg, sc; Dectomax, Zoetis, SP, Brazil) (Passos Nunes, 2023).

To prevent timpanism, sorbitol (80 mg/kg, Sedacol, Calbos, iv or intrarectal) and metoclopramide hydrochloride (1 to 5 mg/kg, Plasil, Sanofi Aventis, im or iv) were used in individual cases (Passos Nunes 2023). If necessary, typhlocentesis would be performed with a 40G needle in the most tympanic region of the abdomen.

**Figure 3:**
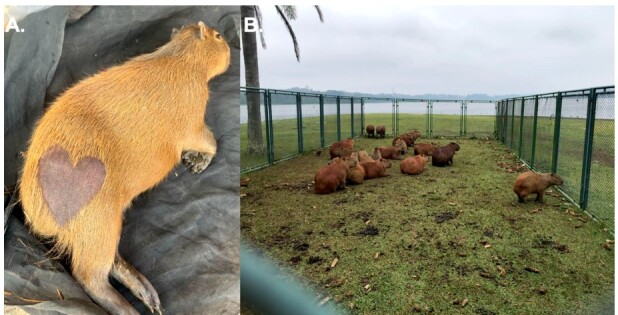
Capybara in the immediate postoperative period (A) and prior release at the same place of capture (B). Photo: AZNunes LTDA.

Animals received subcutaneous microchip at the interscapular region for further follow-up. Additionally, the animals' hindquarters were sheared into a heart shape, aiming to facilitate visual differentiation of sterilized individuals and to foster compassion among local residents (Figure 3A). This measure seeks to mitigate the fear, based on misconceptions, that capybaras might directly transmit bacteria to humans (Passos Nunes, 2023). Postoperative recovery occurred in the same traps in which the animals were initially captured, and the animals were released after complete anesthetic recovery (walking normally, alert and reactive) in the same place of capture (Figure 3B). Post-operative monitoring was conducted through a daily observation over the subsequent 2 weeks.

### Surgical procedures

**Figure 4:**
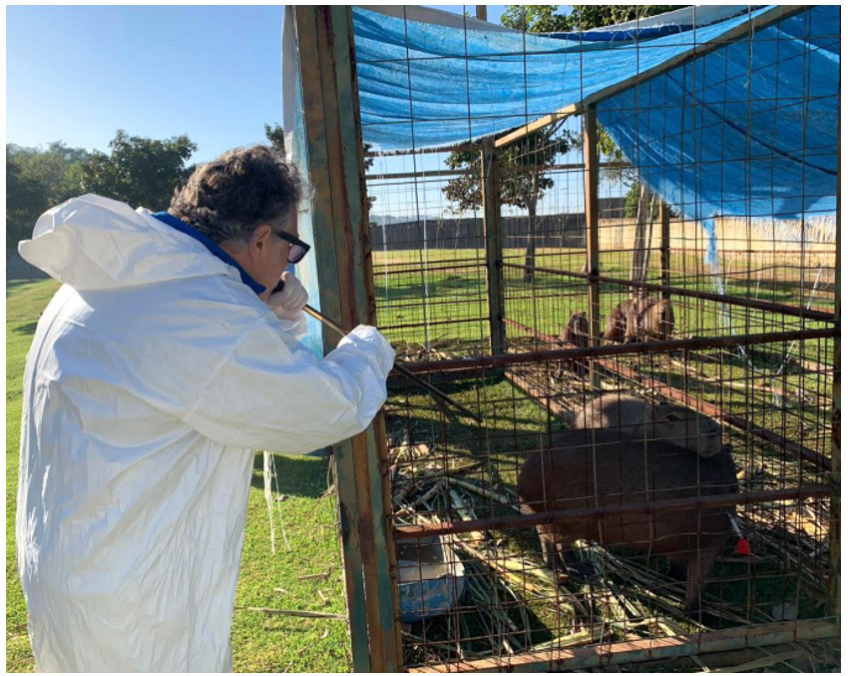
Adapted container for surgery performance.

Surgical procedures were conducted within specially modified containers or rooms situated on the property and in close proximity to the site of capture (Figure 4). Every surgical procedure was executed by a single surgeon, and the animals were pre-operatively prepared in accordance with the methods outlined by Passos-Nunes et al. (2022). The surgical techniques were alternated. The incision size was measured using calipers, and the time was recorded using a digital stopwatch, measured by more than one person.

Perioperative complications and challenges encountered during surgical procedures were carefully documented, emphasizing difficulties in identifying reproductive structures, ensuring sufficient exposure of these structures, preserving vascular integrity, and accurately performing the surgical technique. Post-operative follow-up was feasible solely in recapture scenarios, where a comprehensive evaluation of the surgical incisions was conducted, considering the free-living condition of the animals.

#### Partial salpingectomy

The animals were placed in lateral recumbency (right or left), and the skin incision was made caudally to the last rib, ventral to the sublumbar musculature, between the third intertransverse space and the iliac crest, following the approach suggested by Jorge et al. (2023). The mini-laparotomy incision was performed along a cranio-caudal axis (longitudinal), following the anatomic topography of the ovary, and was extended as necessary to expose the ovary and uterine tube.

After the skin incision, the subcutaneous tissue, cutaneous muscle (m. *panniculus carnosus*), and abdominal structures (transversalis fascia, external abdominal oblique muscle, internal abdominal oblique muscle, transversus abdominis muscle, and parietal peritoneum) were bluntly dissected to access the dorsolateral region. Following digital traction and exposure of the uterine tubes, the mesosalpinx was carefully dissected to allow for the placement of two separate ligatures using 3-0 nylon suture (Shalon Medical, Goiânia, GO, Brazil), maintaining a minimum distance of 2 cm between them. Nylon was chosen over absorbable suture materials to prevent the technique's reversibility, given that the salpingectomy was partial, not total.

**Figure 5:**
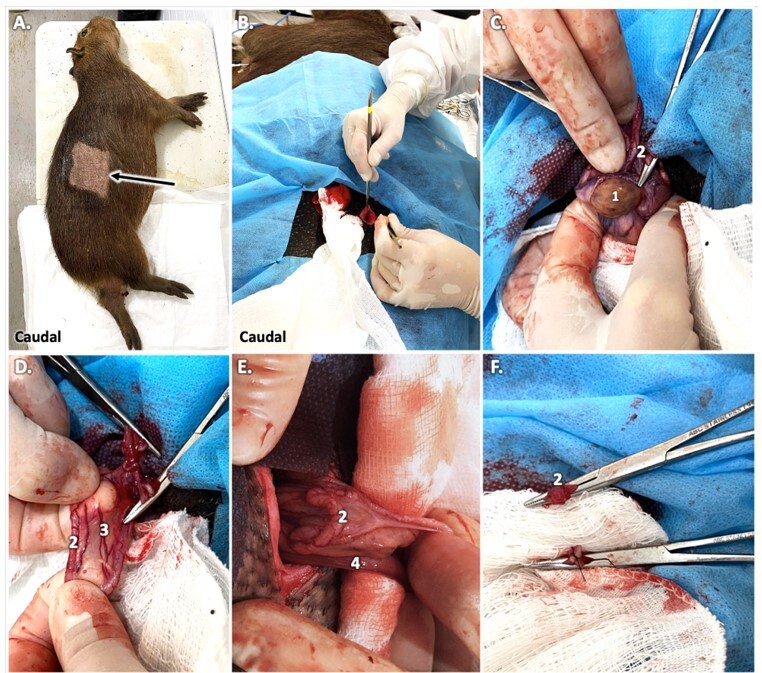
Steps of partial salpingectomy in female capybaras. Animal positioned in lateral recumbency prior skin antisepsis (A); incision over a cranio-caudal axis (B); ovary (1) and uterine tube (2) exposed (clamped) C, notice the vascularized mesosalpinx (3) (D); individualized uterine tube for salpingectomy performance and proximal end of uterine horn (4) (E); uterine tube after ligature and transection (F). Photo: AZNunes LTDA.

**Figure 6:**
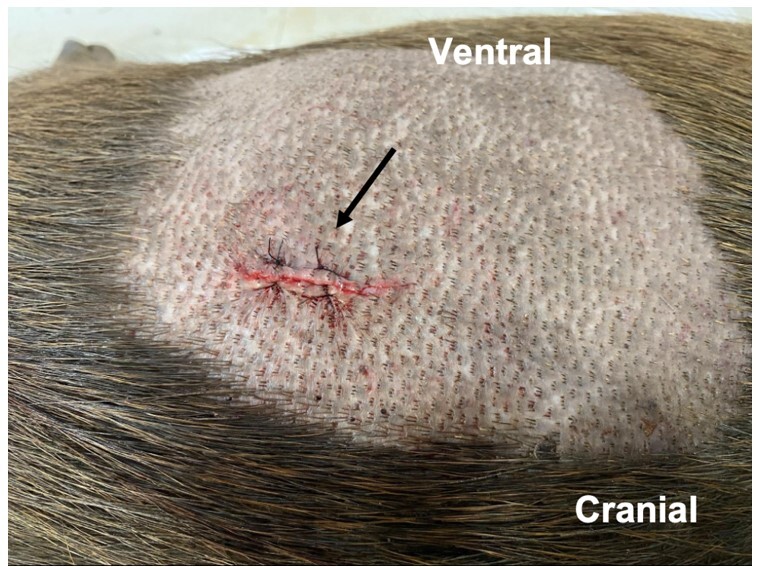
Female capybara in lateral recumbency. Surgical wound appearance in the immediate postoperative period. Photo: AZNunes LTDA.

A segment of at least 1 cm of the uterine tube was removed to prevent recurrence. The ovaries and uterine tubes were then repositioned in the abdominal cavity, and the musculature was sutured en bloc (cruciate suture using nylon 2-0; Shalon Medical, Goiânia, GO, Brazil) (Gaber and Abdel-Wahed, 2015). Subcutaneous suturing was performed using subcuticular suture, while the skin was sutured using a horizontal mattress or cruciate pattern (2-0 or 3-0 nylon thread; Shalon Medical, Goiânia, GO, Brazil) (Gaber and Abdel-Wahed, 2015). In females with a more pendulous abdomen, 2.0 nylon was the preferred choice. Additionally, surgical glue (Tissue Aid TM, 2-Octyl Cyanoacrylate) was applied to the sutured skin. The same procedure was replicated on the contralateral flank (Figures 5 and 6).

#### “Passos Nunes” uterine horn ligature

**Figure 7:**
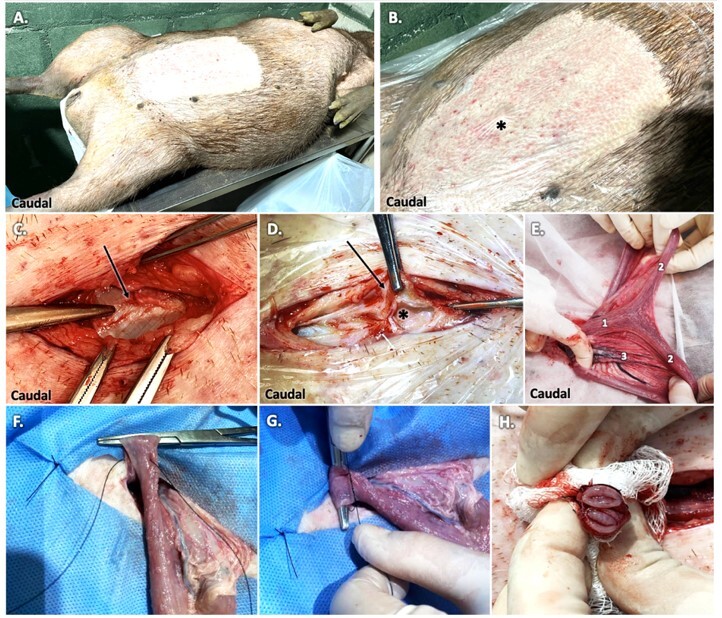
Steps of “Passos Nunes” uterine horn ligature: Female capybara in dorsal recumbency (A) and place of incision caudally to umbilical scar (*) (B); identification (C) and incision of *linea alba* (arrow). The incision might be gentle to avoid accidental urinary bladder or cecum incision when distended. Exposition of the uterine body (1) and uterine horns (2 - right and 2’ - left), note the wide distribution of arteries and veins in the mesometrium (3), favoring hemorrhage when damaged (E). Uterine horn ligature (F, G) and section (H). Photo: AZNunes LTDA.

The surgical procedure was conducted in accordance with the methodology described by Passos Nunes et al. (2022). Briefly, a 3-centimeter incision was made above the pubis. This was followed by the dissection of the *panniculus carnosus* muscle and the incision of the *linea alba* to reveal the peritoneal cavity. In instances where the *linea alba* could not be readily identified, the ventral abdominal musculature was dissected to gain access to the peritoneal cavity. The urinary bladder was then retracted to expose the uterine horn. Crile hemostatic forceps were clamped on the cranial end of the uterine horn, forming a loop, near the uterine tube. A transfixation suture was placed under the hemostatic forceps using nylon 2.0 or 3.0 suture thread, depending on the size of the uterine horn (Figure 7 [Fig gf08] to 9), effectively obstructing the uterine horn. The distal end of the ligated uterine horn was subsequently partially incised, serving as an additional measure to obstruct communication between the cranial and distal ends beyond the transfixation site. The abdominal muscles were then sutured using a cross-mattress pattern, and the *panniculus carnosus* muscle was co-sutured along with the subcutaneous tissue using a subcuticular suture. Finally, surgical glue was applied to the sutured skin.

**Figure 9:**
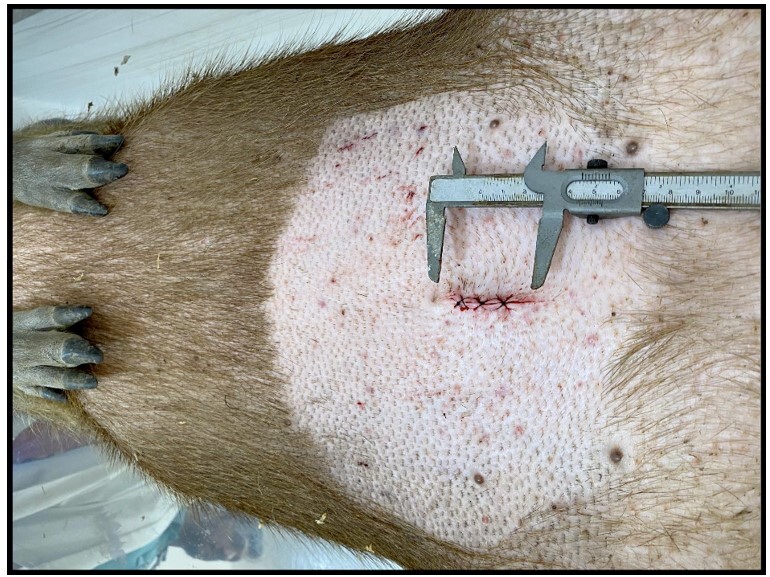
Female capybara in dorsal recumbency. Surgical wound appearance in the immediate postoperative period. Photo: AZNunes LTDA.

**Figure 8 gf08:**
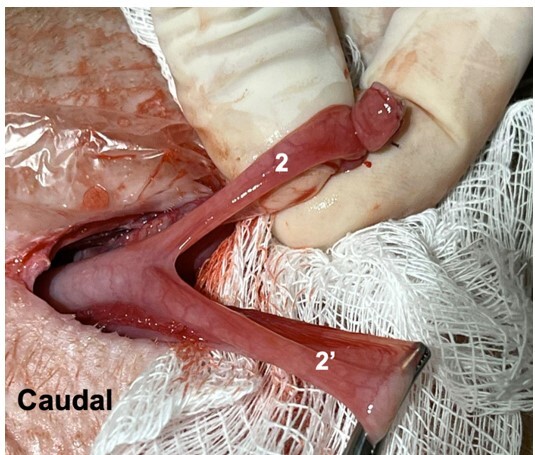
Ligature performed on the right uterine horn (2) and to be done on the left (2’) Photo: AZNunes LTDA.

### Statistical analysis of time

The sample size was determined using the G*Power software (Faul et al., 2007) to suit the data, utilizing the total surgical time (measured in minutes)—spanning the period from the initiation of the skin incision to the completetion of skin closure (Silva et al., 2012; Acharya et al., 2016; Powell et al., 2017; Nguyen et al., 2019)—as the main variable. A preliminary study yielded an effect size of 1.611621 which, at α = 5% and 1-β = 80%, suggested the inclusion of 8 animals per group. To compare the differences between methodologies, an Independent Samples t-test (Equal Variance) was employed, preceded by the Shapiro-Wilk normality test and F-test for equality of variances, both of which confirmed a normal distribution and equal variances. The results thus obtained were used in the concluding statistical analysis conducted using the R software).

## Results

**Figure 10:**
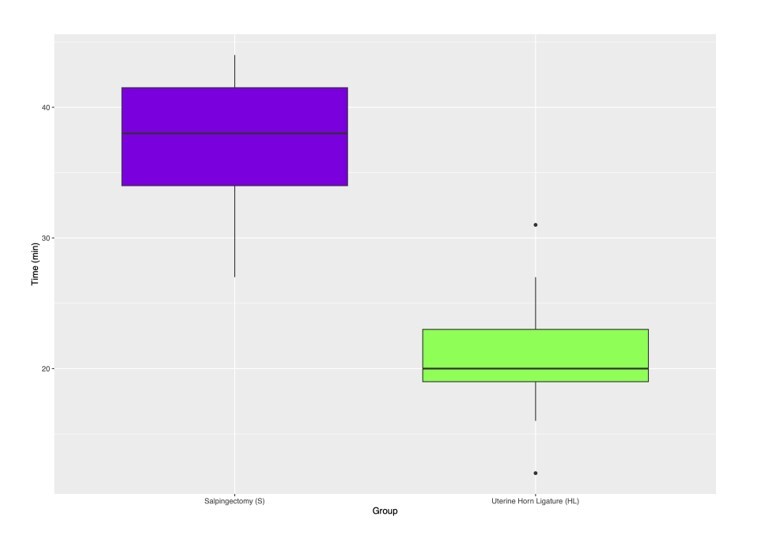
Difference of operative time between HL and S groups.

Data about to the transoperative time (in minutes) were comprehensively analyzed, with findings including the mean, median, standard deviation, and interquartile range (Table 1 and Figure 10). The “p” value and the confidence interval are detailed in Table 2, indicating “HL” as the fastest technique, with differences ranging from 11 minutes and 06 seconds to 21 minutes and 06 seconds (95% CI).

**Table 1:** Comparison of time (min) between HL and S groups.

**Group**	**Mean (min)**	**Median**	**Standard** **deviation**	**IQR**
HL	21:05	20:00	5:09	4:00
S	37:06	38:00	5:48	7:30

IQR: interquartile range; HL: uterine horn ligature group; S: partial salpingectomy group.

**Table 2:** “p”value and confidence interval for t test in relation to the variable time.

	**p value**	**CI (95%)**
Total operative time	2.087 * 10^(-6)	11.01 to 21.01

p: significance; CI; confidence interval.

**Figure 11:**
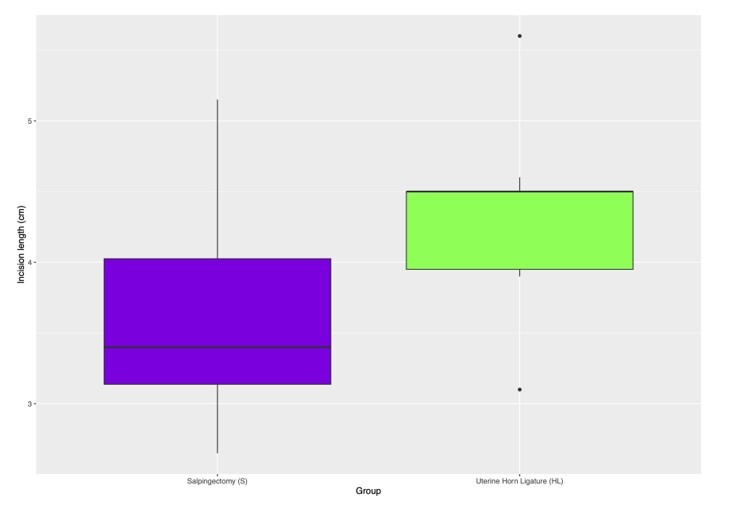
Difference between incision length on HL (uterine horn ligature) and S (partial salpingectomy) groups, showing no practical relevance.

Despite a statistically significant variance in the incision length between the two techniques, the practical impact of this difference appears negligible. Specifically, the average disparity in incision length was minor (0.71 cm), with the minimal boundary of the confidence interval being 0.06 cm, a margin considered clinically inconsequential. Detailed statistical analyses, including mean, median, standard deviation, and interquartile range for this aspect, are depicted in Table 3 and Figure 11. The “p” value and the confidence interval for the hypothesis test are described in Table 4.

**Table 3:** Comparison of incision length (cm) between HL and S groups.

**Group**	**Mean (cm)**	**Median**	**Standard** **deviation**	**IQR**
HL	4.327	3.4	0.618	0.550
S	3.615	4.5	0.812	0.887

IQR: interquartile range; HL: uterine horn ligature group; S: partial salpingectomy group.

**Table 4:** “p” value and confidence interval for t test in relation to the incision length.

	**p value**	**CI (95%)**
Incision length (cm)	0.03465	0.06 to 1.37 cm

p: significance; CI; confidence interval.

The perioperative phase was notably devoid of complications attributable to the surgical interventions. To address instances of tympany, sorbitol was administered to three subjects within the HL and to one animal in the S groups. In an effort to preempt gastrointestinal complications, metoclopramide was provided to two females in the S group. A singular case of mesosalpinx bleeding occurred in the S group during the uterine tube ligature/transection process, which was efficiently resolved through immediate hemostatic intervention.

The partial salpingectomy procedure permitted the easy exposure and traction of the ipsilateral ovary and uterine tube for tubal ligature and partial resection. However, this approach did not provide access to the contralateral uterine tube, necessitating bilateral incisions. Consequently, this required changing in the patient's position during surgery, antisepsis of the contralateral flank, and replacement of contaminated disposable materials, increasing in the total operative time. Additionally, it was not possible to expose the uterine horns through the incision, which had been sufficient for exposing the ovary and uterine tube.

Conversely, the Passos-Nunes uterine horn ligature, using a post-umbilical celiotomy along the *linea alba*, necessitated a mini-laparotomy incision and provided superior exposure to the uterus. However, it did not allow for the identification and exposure of the ovaries and uterine tubes. This procedure did not require changing the patient's position or using of new disposable materials during surgery. During the midline incision, the *linea alba* was difficult to discern due to the robust fixation of subcutaneous tissue; therefore, palpation was essential for its identification. Both surgical approaches (flank and midline) facilitated minimal visceral manipulation of the abdominal organs.

**Figure 12:**
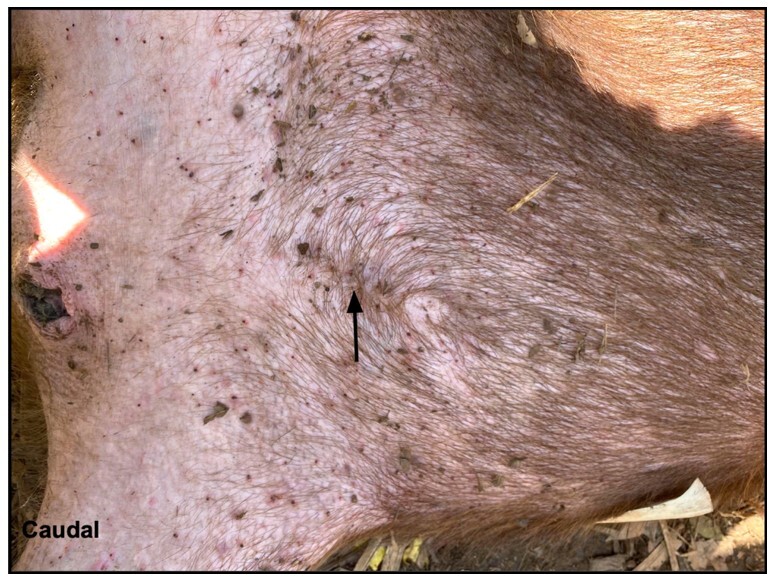
Female capybara recaptured after 2 years of surgery. Note that surgical scar is hardly identified. Photo: AZNunes LTDA.

No instances of mortality or postoperative complications were documented, with all females remaining alive until the last capture in July 2022, during which 14 females were successfully recaptured. Observations from females recaptured one to three months post-surgery indicated complete healing of the surgical wounds (Figure 12), irrespective of whether the flank or midline surgical approaches were employed.

## Discussion

The escalating urban incursion of capybaras in Brazil poses a significant public health challenge, primarily due to their capacity to transmit BSF. Addressing the burgeoning capybaras population necessitates a holistic approach, aligning with the principles of One Conservation (Pizzutto et al., 2021), and mandates collaborative efforts across various institutions for effective resolution.

Central to mitigating this issue is the implementation of reproductive management and population control strategies, including the application of contraceptive techniques (Passos-Nunes et al., 2022). While contraceptive vaccines for male capybaras have shown promising results (Rosenfield et al., 2019b), their practical application is hindered by market unavailability. Conversely, surgical techniques have proven effective and offer various procedural options (Ferraz, 2013; Rodrigues et al., 2017; Passos Nunes et al., 2020; Passos-Nunes et al., 2022; Yanai et al., 2022).

In the state of São Paulo, government agencies have endorsed surgical sterilization methods that preserve the gonads (São Paulo, 2016; Rosenfield et al., 2019c). This endorsement underscores the importance of meticulous care across pre-operative, intra-operative, and post-operative phases, especially considering the free-ranging habitats of these animals, to ensure swift recovery.

This study evaluates two surgical sterilization methods for female capybaras: the “Passos Nunes” uterine horn ligature (LH) and partial salpingectomy (S). The focus was on minimizing intestinal manipulation and simplifying uterine horn exposure in both methods to prevent postoperative ileus, a consideration crucial for pregnant females requiring hysterotomy or hysterectomy (Passos Nunes et al., 2020; Passos Nunes, 2023). Other evaluated parameters included transoperative time and incision length, with the hypothesis that in a single approach incisions might reduce perioperative challenges and surgical difficulties.

The comparison of these distinct surgical approaches acknowledges potential limitations due to their differing methodologies. However, the study posits that shorter total management times for wild animals—which encompass physical restraint, chemical restraint, transoperative period, anesthetic recovery, and release—may correlate with improved prognoses. Given that the total transoperative time directly impacts surgical efficacy, comparing the transoperative durations of both techniques is deemed essential for a comprehensive evaluation.

Contrasting with the uterine horn ligature technique, bilateral partial salpingectomy (BPS) requires an extended transoperative duration, potentially elevating the risk of postoperative complications (Ando et al., 2005; Hardy et al., 2014; Cheng et al., 2017; Duchman et al., 2017; Bohl et al., 2018). This increased operative time is attributed to the need to change the animal's position (decubitus), conduct new antisepsis of the surgical area, and replace disposable materials for the contralateral laparotomy. This observation aligns with findings by Janssens and Janssens (1991), who noted a similar challenge with bilateral flank ovariectomy in female dogs, wherein the procedural complexity contributed to extended surgery times. To mitigate the prolonged duration associated with BPS, employing bipolar cautery (Nguyen et al., 2019) or titanium hemostatic clips (Lawrie et al., 2015; Yanai et al., 2022) for tubal occlusion and hemostasis can significantly reduce operative time. Additionally, laparoscopic tubal ligation using titanium hemostatic clips emerges as a promising alternative (Deco-Souza et al., 2024).

A hemorrhage of the mesosalpinx, occurring during the ligature/transection of the uterine tube in a subject from group “S,” represented the sole documented incident; this was, however, efficiently managed. In contrast, ligature of the uterine horn could precipitate profuse bleeding due to the inadvertent rupture of one or more branches of the uterine artery, which are distributed throughout the mesometrium. It is noteworthy that, based on our experience, multiparous females, who possess uterine horns that are both broader and more vascularized, face an elevated risk of hemorrhage during surgical procedures. It is important to highlight that the surgeon had advanced training in the HL technique, which was applied to more than 100 females during reproductive management in regions at risk of BSF. Her experience, while indicative of a high level of surgical expertise, could also be construed as a potential bias in evaluating the technique's efficacy and safety.

Concerning surgical methodology, the ipsilateral ovary and uterine tube could be conveniently exposed and tractioned for uterine tube ligature and partial resection via flank mini-laparotomy, as described by Jorge et al. (2023) and Pradere et al. (2006), but not the uterine horns. The contralateral ovary and uterine tube could not be reached, and a bilateral approach was necessary (Yanai et al., 2022; Jorge et al., 2023).

Conversely, ligature of the uterine horn via post umbilical celiotomy along the *linea alba* required only one mini-laparotomy incision and provided adequate exposure of the uterus. This surgical approach obviated the need for changes in the animal's position (decubitus) or disposable materials during the procedure. Due to the friable consistency of capybara muscles, caution must be taken when inciting the *linea alba* to prevent unintended incision of the urinary bladder. This tissue characteristic also influenced the surgical decision to suture the abdominal muscles in the flank region en bloc rather than individually suturing each muscle layer, especially when the *linea alba* was indistinguishable, and the musculature was dissected bluntly. The difficulty in identifying the *linea alba* through the midline incision was further compounded by the robust adhesion of the subcutaneous tissue, presenting an additional challenge in the surgical procedure.

Concerning the impact of the incision axis on the visibility of reproductive organs and the efficacy of the surgical technique, Yanai et al. (2022) chose to conduct a partial salpingectomy through a dorso-ventral (transverse) incision in the flank. This approach is akin to the technique utilized by McGrath and Davis (2004) during ovariectomies in bitches via the flank. Conversely, Janssens and Janssens (1991), Ferreira et al. (2015), and Bester et al. (2021), preferred the craniocaudal (longitudinal) incision axis, the same orientation employed in the current study. Both incision axes provide sufficient exposure to the reproductive organs. Expanding the surgical incision along a transverse axis could allow for the visualization of the contralateral uterine tube.

However, enlarging the incision to facilitate manipulation of the cecum increases the likelihood of postoperative ileus (Boeckxstaens and De Jonge, 2009; Leslie et al., 2011; Hellstrom et al., 2021), a concern especially pertinent given the cecum's extensive positioning within the abdominal cavity (Rosenfield et al., 2020; Jorge et al., 2023) and the dorsal topography of the ovary in capybaras, which necessitates the manipulation of the cecum to achieve exposure of the contralateral uterine tube. Considering the high risk of postoperative tympanites, a frequent complication in capybaras (Rosenfield et al., 2019a), cecal manipulation should be minimized during surgery. This consideration underscores the utility of a post-umbilical incision, as utilized in the Passos Nunes technique, to mitigate these risks.

While the benefits of employing a transverse incision axis, such as enhanced tissue regeneration and reduced postoperative complications compared to a longitudinal axis in human surgeries, are documented (Grantcharov and Rosenberg, 2001), this study did not explore these distinctions. For optimal exposure of the uterine horns and minimal intestinal manipulation, the ventral abdominal approach may be preferable to the flank approach, especially in pregnant females undergoing hysterotomy (Passos-Nunes et al., 2022).

Regarding visceral manipulation, the observed impairment of intestinal motility and subsequent intestinal tympanism point to a potential iatrogenic factor, aligning with Rosenfield et al. (2020) findings after administering ketamine and dexmedetomidine to capybaras. The capacity of ketamine (Zholos et al., 2023), α_2_-adrenoceptor agonists (Blandizzi, 2007), and opioids (Kurz and Sessler, 2003) to induce tympany is acknowledged, attributed to their inhibitory effects on intestinal motility and secretion. Nonetheless, our adoption of multimodal analgesia (Coutens et al., 2020) aimed to mitigate such risks. Factors contributing to the low occurrence of tympanism-related complications in our study, based on the author's personal experience, include a 12-hour fasting period prior to the procedure and the administration of an α-adrenergic antagonist (yohimbine), which is known to improve gastrointestinal motility (Tanila et al., 1993), coupled with a single dose of an opioid administered prior to surgery.

The choice of xylazine over other more specific α-adrenergic agonists like detomidine or dexmedetomidine was motivated by its cost-effectiveness, widespread commercial availability, and proven safety profile in capybaras when administered appropriately. Furthermore, xylazine's potent analgesic properties have been validated in capybaras (Nishiyama et al., 2006) and employed by various veterinary teams (Nishiyama et al., 2006; King et al., 2010; Rego et al., 2010; S. Monsalve-Buritica et al., 2013; Hokamp et al., 2020; Passos Nunes et al., 2020; Neves et al., 2022; Passos-Nunes et al., 2022; Caixeta et al., 2024), underscoring its appropriateness in capybara anesthesia, provided it is used correctly.

While this study incorporated metoclopramide hydrochloride to manage tympanism in animals, existing literature does not substantiate its efficacy in shortening the duration of postoperative ileus in humans or rodents (Bungard and Kale‐Pradhan, 1999; Traut et al., 2008; Boeckxstaens and De Jonge, 2009). Conversely, in Arabian horses, an association between the administration of metoclopramide and increased cecal and colonic contractions has been documented (Beder et al., 2020).

Implementing effective strategies for mass sterilizing capybaras in their natural habitat presents a significant challenge, particularly given this species' high susceptibility to stress and complications from post-surgical wound injuries. Adopting a ventral abdominal surgical approach emerges as an optimized strategy due to its proven efficacy in protecting the surgical wound. This aspect is critically important, considering the typical aggressive behavior among capybaras and the unique configuration of the incision. Such a characteristic becomes particularly relevant when the animals are reintegrated into their natural environment after anesthesia recovery, thus enabling remote post-operative monitoring.

In contrast to traditional salpingectomy techniques, which require bilateral incisions and may inadvertently be performed on pregnant females, uterine horn ligation presents itself as a viable alternative. This technique eliminates the need for pre-operative ultrasonography to confirm pregnancy, thereby reducing the risk of unplanned births and complications associated with the use of anesthetics, which could result in fetal or maternal death.

However, evidence in the scientific literature suggests that a lateral surgical approach can significantly improve wound healing (McGrath and Davis, 2004; Reece et al., 2012; Stepanov, 2020) and enhance the efficacy of wound monitoring after the animal's release. The data presented in this study aim to contribute to the refinement of population control practices through contraceptive surgical methods applied to capybaras in free-living contexts, ultimately seeking to promote the well-being of this species.

## Conclusion

The Passos-Nunes uterine horn ligature emerges as a superior alternative for open surgical sterilization, as evidenced by its expedited procedure compared to traditional uterine ligature in female capybaras within this study. This technique is characterized by minimal intestinal manipulation and a reduced duration of the surgery relative to tubal procedures while also facilitating the performance of hysterotomy or hysterectomy. Given the data presented, it is reasonable to conclude that uterine horn ligature is a recommended strategy in regions afflicted by Brazilian spotted fever, contributing to the long-term mitigation of this disease. This recommendation is predicated on the method's efficiency and potential to decrease the vector population through sterilization, thereby aiding in the control of Brazilian spotted fever transmission.
